# Cross-cultural adaptation of the milestones project in otolaryngology for the brazilian reality

**DOI:** 10.1590/0100-6991e-20233570-en

**Published:** 2023-07-19

**Authors:** NEILOR FANCKIN BUENO MENDES, IZABEL CRISTINA MEISTER COELHO

**Affiliations:** 1 - Faculdades Pequeno Príncipe, Mestrado Stricto Sensu em Ensino nas Ciências da Saúde - Curitiba - PR - Brasil; 2 - Faculdades Pequeno Príncipe, Coordenação do Mestrado Stricto Sensu em Ensino nas Ciências da Saúde - Curitiba - PR - Brasil

**Keywords:** Otolaryngology, Internship and Residency, Educational Measurement, Competency-Based Education, Validation Study, Otolarrinolaringologia, Internato e Residência, Avaliação Educacional, Educação Baseada em Competências, Estudo de Validação

## Abstract

**Introduction:**

competency-based medical education is well established, but there is a worldwide shortage of instruments capable of assessing these doctors in training.

**Objective:**

to validate the instrument The Otolaryngology - Head and Neck Surgery Milestone Project for use in Residency Programs in Otorhinolaryngology in Brazil.

**Method:**

The study had 5 stages. In stage I, two independent translations of the Milestones Project in otorhinolaryngology were carried out. In step II, a synthesis of the translations was performed. Subsequently, the competencies required by the Brazilian Association of Otorhinolaryngology for training otorhinolaryngologists in Brazil were added. In step III, a back-translation of the instrument was carried out and sent to the original authors. Then, the instrument was sent to be evaluated by a committee of 8 experts. In stage IV, each expert made comments about each of the items, and after analyzing the suggestions, a new instrument was created. In stage V, this instrument was sent for evaluation by otorhinolaryngologists across the country.

**Results:**

after translations and expert evaluation, an instrument with 19 items was created. The instrument was submitted to analysis by Otorhinolaryngologists from all over Brazil. Acceptance percentages were: applicability (99.25%), reliability (99.5%), reproducibility (98.6%), reliability (93.84%), relevance (93.15%).

**Conclusion:**

the created instrument was considered applicable, reproducible, relevant, reliable and trustworthy, presenting content validity.

## RESUMO

**Introdução:**

a formação médica baseada em competências é bem estabelecida, mas há uma escassez mundial de instrumentos capazes de avaliar esses médicos em formação.

**Objetivo:**

validar o instrumento The Otolaryngology - Head and Neck Surgery Milestone Project para uso nos Programas de Residência em Otorrinolaringologia no Brasil.

**Método:**

o estudo conteve 5 etapas. Na etapa I foram realizadas duas traduções independentes do Projeto Milestones em otorrinolaringologia. Na etapa II, foi realizada uma síntese das traduções. Posteriormente foram acrescentadas as competências que a Associação Brasileira de Otorrinolaringologia exige para formação do otorrinolaringologista no Brasil. Na etapa III foi realizada uma retro tradução do instrumento e enviado para os autores originais. Em seguida, o instrumento foi enviado para ser avaliado por um comitê com 8 experts. Na etapa IV, cada expert fez comentários acerca de cada um dos itens, e após análise das sugestões foi criado um novo instrumento. Na etapa V, este instrumento foi enviado para apreciação de otorrinolaringologistas de todo o país.

**Resultados:**

após as traduções e apreciação dos experts foi criado um instrumento com 19 itens. O instrumento foi submetido à análise de Otorrinolaringologistas de todo Brasil. As percentagens de aceitação foram: aplicabilidade (99,25%), fidedignidade (99,5%), reprodutibilidade (98,6%), confiabilidade (93,84%), relevância (93,15%).

**Conclusão:**

o instrumento criado foi considerado aplicável, reprodutível, relevante, confiável e fidedigno, apresentando validade de conteúdo.

## INTRODUCTION

After graduating, a considerable number of physicians seek specialization through selections for medical residency. Medical residency in Brazil is regulated by Law 6,932/1981 as a Lato Sensu postgraduate teaching modality aimed at complementary medical training, being considered the gold standard of Brazilian medical specialization^1^.

To allow residents to reach the learning goals listed for each specialty, the institutions that offer the programs must provide conditions for the integrated and harmonious development of skills related to knowledge and practice^1^. 

Over the past 13 years, the construction of pedagogical projects for Residency programs has been outlined by the CNRM Resolution 2/2006^3^, which establishes priority themes for the theoretical content of each program and the minimum practical workload in each field of education.

Upon completion of the residency program, the specialist physician should have the necessary skills to develop his/her profession, and the institution is responsible for evaluating and monitoring the entire acquisition process.

According to the Brazilian Association of Otolaryngology and Cervico-Facial Surgery, the skills necessary for training a resident in otolaryngology are mastering anatomy, pathophysiology, interpretation of exams, and clinical and surgical treatment in the areas of rhinology, neck, laryngology and trachea, oral cavity and salivary glands, otoneurology, otology, sleep medicine, occupational otolaryngology, and facial and maxillofacial plastic surgery^4^. 

In Brazil, there is no specific instrument published for assessing the evolution in the acquisition of competences by the resident in otolaryngology. One way other specialties have found to solve this problem is to adapt instruments already available in other languages, such as the Milestone Projects.

The Milestone Projects of the North American Accreditation Council for Undergraduate Medical Education (ACGME) is one of the projects being developed with the American Specialist Societies for the semiannual/annual follow-up of milestones in the development of knowledge, skills, and attitudes during training of physicians in the most diverse specialties, including otolaryngology^5^. 

Faced with the challenge of evaluating medical skills in medical residency, and especially in otolaryngology, the aim of this study is to validate the instrument The Otolaryngology - Head and Neck surgery Milestone Project for use in Otolaryngology residency programs in Brazil.

## METHODS

This study is a methodological study that aims to investigate the gathering, organizing, and analyzing of data obtained during the development and validation of an evaluation instrument. Methodological studies aim to investigate methods for collecting and organizing data, such as development, validation, and evaluation of research tools and methods, which favors conducting investigations with accentuated rigor^6^. 

The study was approved by the Ethics in Research Committee under number 4,282,391. The development of the instrument was based on the model proposed in the AMEE guide^7^: 

[Fig f1]

**Figure f1:**
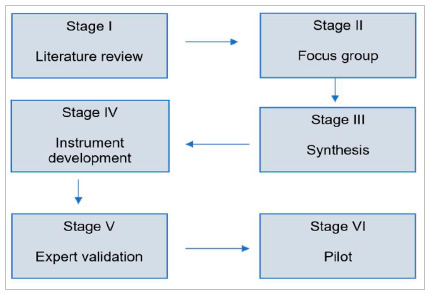

Each of the steps will be described below, followed by the results obtained in each of them.

### Step I - Literature review

PubMed, Medline and Google Scholar databases with the following terms: cross cultural adaptation, Milestones, otolaryngology, Portuguese, acgme, otorrinolaringologia, avaliação, Brasil, and residentes.

### Stage II - Focus group

In the absence of documents available in the Brazilian literature to evaluate competences in otolaryngology, we searched the competences required by the ABORLCCFF (Brazilian Association of Otolaryngology and Cervico-Facial Surgery), and separated them by means of tables, over the three years of training for otolaryngologists.

Then, we obtained the instrument The Otolaryngology - Head and Neck surgery Milestones Project and analyzed its structure and contemplated competences.

With all the necessary competencies for the training of the general otolaryngologist according to ABORLCCF divided by year of training, and with the document Milestones in otorhinolaryngology, we compared the two documents. 

### Stage III - Synthesis

After seeking and receiving authorization from the original author of the Milestones in Otolaryngology, we conducted two translations of the document. Both translators had Portuguese as their mother tongue. One had domain knowledge about the concepts addressed in the instrument (health area), and the other had not.

Each translator performed a separate translation, producing instruments A1 and A2.

Then, we conducted a synthesis of the translations, creating instrument B. To this end, we constructed a table with the differences between the two translations A1 and A2. The table contained three columns: the first column had the text from the regular translator, the second had the text of the translator from the health area, and the third had the chosen option. To arrive at the chosen option, a consensus meeting was held between the translators and the authors.

### Stage IV - Instrument development

After creating instrument B, with the syntheses of the translations, the authors revised it in view of the competences required by the Brazilian Association of Otolaryngology and Cervico-Facial Surgery (ABORLCCF). The skills listed by ABORLCCF that were missing in instrument B were added, thus creating instrument C.

### Step V - Experts validation

For the evaluation stage of document C, we invited 12 experts. The selection criteria were being an otolaryngologist member of the ABORLCCF and being a preceptor of a residency program or medical specialization in otolaryngology, with at least 3 years of experience.

The experts were numbered following alphabetical order, and a table was created with a summary of the opinions issued by them on each of the items.

We analyzed all the observations made by the experts, always considering the competences required by the ABORLCCF (Brazilian Association of Otolaryngology and Cervical Facial Surgery), adapting and restructuring the instrument into instrument D.

We then sent this new instrument back for the appreciation of the experts and this time all agreed that the instrument was complete and comprehensive, thus creating the instrument D.

### Stage VI - Pilot

In this step, we adapted instrument D to the Survey Monkey® software in the form of a semi-structured questionnaire in which each subspecialty item within otolaryngology should be evaluated in terms of accuracy, reproducibility, reliability, and applicability.

Thus, the instrument developed was submitted to the judgment of otolaryngologists throughout Brazil, to quantify its acceptance. We sent a link with an invitation to otolaryngologists across the country to evaluate the final instrument created. The criterion for sending the questionnaire was to be an otolaryngologist and member of ABORLCCF. We analyzed the content validation, considering the adequacy percentages based on the professionals’ responses.

For the analysis, the acceptance percentages were calculated for each question in each of the categories and a Confidence Interval was also presented for this percentage. As a reference, we considered a percentage of acceptance of 90% as good, using a significance level of 0.05, which is equivalent to a confidence of 95%.

## RESULTS

With the increase in the number of multinational and multicultural research projects, the need to adapt health assessment instruments has grown rapidly^8^. Many of these questionnaires and assessment instruments were developed in English^9^.

The adaptation of questionnaires and assessment instruments in the health area for use in other countries, cultures, or languages requires specific methods to achieve equivalence between the original source and the desired version. For the original instruments to be valid in realities different from the ones they were created, they must not only be literally translated, but also undergo rigorous and systematic adaptation to maintain their validity and concepts across different cultures^10^.

In the absence of documents available in the Brazilian literature to evaluate competences in otolaryngology, we searched and tabulated the competences required by the ABORLCCFF (Brazilian Association of Otolaryngology and Cervico-Facial Surgery) over the three years of training for otolaryngologists.

Then, we obtained the instrument The Otolaryngology - Head and Neck surgery Milestones Project and analyzed its structure and contemplated competences.

### Step I - Literature review

After carrying out a literature review, we found no adaptation of the Milestones Project to Brazilian Portuguese, and there is no specific instrument published in which it is possible to monitor, in a continuous and progressive way, the evolution of the skill acquisition by the residents/specialists in otolaryngology.

### Stage II - Focus group

After analysis, we concluded that the Milestones document for otolaryngology had most of the competencies required by the ABORLCCF, and presented a structure that favored the evaluation of each of the residents/specialists during their formative years. Thus, we decided to use the Milestones in Otolaryngology as a reference for the creation of the new instrument.

### Stage III - Synthesis

To summarize the translations, we created tables and analyzed the differences between translators. In all, 119 divergences were raised, which we divided into three categories for better classification:

a) Category 1 (79 items): both translations were correct;
b) Category 2 (18 items): both translations were correct, but one of the medical terms was more suitable for use in Brazil; and
c) Category 3 (19 items): the translation with a medical term was correct, since the literal translation did not apply.

Category 1 presented 79 divergences. In this case, both translations were correct, but the one that proved to be more suitable for an evaluation instrument was chosen. Thus, “develops positive relations” was chosen instead of “develops positive relationships”; “demonstrates basic understanding” rather than “demonstrates limited knowledge”; “ability to approach complications” instead of “ability to manage complications”; and “conducts appropriate assessments for patient management” rather than “ensures appropriate consultations for patient management”.

In the second category, with 18 divergences, both translations would be suitable for use, however, those performed by a translator belonging to the health area showed a more adequate semantics for the construction of an evaluation instrument in otolaryngology: “cancers” instead of “malignant disease”; “understands the need for a long-term follow-up plan” instead of “understands the need for a long-term surveillance plan”; “recognizes endoscopic landmarks” instead of “recognizes endoscopic surgical landmarks”.

In the third category, in all 19 divergences found, the translation of the translator from the health area was chosen, since the translation without prior knowledge of technical terms was not adequate. Citations such as “facial skeleton”, “sinonasal pathology”, “elevates nasal mucosa flaps”, “Eustachian tube”, and “post-auricular incision”, were substituted by “facial bones”, “nasosinusal pathology”, “elevates septal mucosa flap”, “auditory tube”, and “retroauricular incision”.

### Stage IV - Development of the instrument

The final instrument C contained 17 items encompassing the subtopics within otolaryngology. Each of the items was divided into five levels that comprised the expected evolution of the resident/specialist during their formative years:

a) Level 1: Competencies expected for a resident who has just joined or is in the first months of residency;
b) Level 2: The resident is advancing and demonstrates additional skills but is not yet performing at an average level in the residency;
c) Level 3: The resident continues to advance and further milestones, including most of the targeted skills;
d) Level 4: The resident has advanced and now substantially demonstrates the skills targeted for the residency. This level is designed as a graduation objective; and
e) Level 5: The resident has progressed beyond established performance goals for the residency.

### Step V - Experts validation

In this step, eight experts agreed to answer the instrument analysis questionnaire. Six provided written considerations about the items, and two completely agreed with the instrument’s content. The experts’ experience with teaching residents ranged from three to 30 years, with a mean of 10 and median of 12 years. Of the 17 items evaluated, only four did not receive any observation. Regarding clarity and pertinence, 100% of the experts positively evaluated all items. In the final assessment, all experts positively evaluated the item “the concept was adequately covered”, while the item “all items were included” received a negative response from only one expert, since he observed that the themes related to “dizziness” and “tinnitus” were not completely covered in instrument C. Thus, two new topics with suggested themes were added, restructuring instrument C to instrument D, with 19 items.

### Stage VI - Pilot

Fifty-seven otolaryngologists agreed to participate in this stage and answered it completely. 

Table 1 presents the frequencies and percentages of responses as deemed adequate and the confidence intervals for these percentages.

**Table 1:** Percentages and confidence intervals for question adequacy.

	Questions	Accuracy	Reproducibility	Reliability	Applicability	Relevance
Q01	Salivary Illness	57 (100%) (93.7%; 100%)	56 (98.2%) (90.7%; 99.7%)	56 (98.2%) (90.7%; 99.7%)	56 (98.2%) (90.7%; 99.7%)	56 (98.2%) (90.7%; 99.7%)
Q02	Aerodigestive Tract Injury	57 (100%) (93.7%; 100%)	57 (100%) (93.7%; 100%)	57 (100%) (93.7%; 100%)	57 (100%) (93.7%; 100%)	57 (100%) (93.7%; 100%)
Q03	Sleep and Breathing Diseases	57 (100%) (93.7%; 100%)	57 (100%) (93.7%; 100%)	57 (100%) (93.7%; 100%)	57 (100%) (93.7%; 100%)	57 (100%) (93.7%; 100%)
Q04	Facial Trauma	56 (98.2%) (90.7%; 99.7%)	55 (96.5%) (88.1%; 99.0%)	56 (98.2%) (90.7%; 99.7%)	55 (96.5%) (88.1%; 99.0%)	56 (98.2%) (90.7%; 99.7%)
Q05	Rhinosinusitis	57 (100%) (93.7%; 100%)	57 (100%) (93.7%; 100%)	57 (100%) (93.7%; 100%)	57 (100%) (93.7%; 100%)	57 (100%) (93.7%; 100%)
Q06	Nasal Deformity	56 (98.2%) (90.7%; 99.7%)	55 (96.5%) (88.1%; 99.0%)	56 (98.2%) (90.7%; 99.7%)	56 (98.2%) (90.7%; 99.7%)	55 (96.5%) (88.1%; 99.0%)
Q07	Chronic Ear	56 (98.2%) (90.7%; 99.7%)	56 (98.2%) (90.7%; 99.7%)	56 (98.2%) (90.7%; 99.7%)	57 (100%) (93.7%; 100%)	56 (98.2%) (90.7%; 99.7%)
Q08	Pediatric Otitis Media	57 (100%) (93.7%; 100%)	57 (100%) (93.7%; 100%)	57 (100%) (93.7%; 100%)	57 (100%) (93.7%; 100%)	57 (100%) (93.7%; 100%)
Q09	SADS	56 (98.2%) (90.7%; 99.7%)	56 (98.2%) (90.7%; 99.7%)	56 (98.2%) (90.7%; 99.7%)	57 (100%) (93.7%; 100%)	55 (96.5%) (88.1%; 99.0%)
Q10	Auditory Loss	56 (98.2%) (90.7%; 99.7%)	56 (98.2%) (90.7%; 99.7%)	56 (98.2%) (90.7%; 99.7%)	57 (100%) (93.7%; 100%)	56 (98.2%) (90.7%; 99.7%)
Q11	Dizziness	56 (98.2%) (90.7%; 99.7%)	56 (98.2%) (90.7%; 99.7%)	56 (98.2%) (90.7%; 99.7%)	57 (100%) (93.7%; 100%)	56 (98.2%) (90.7%; 99.7%)
Q12	Tinnitus	57 (100%) (93.7%; 100%)	57 (100%) (93.7%; 100%)	57 (100%) (93.7%; 100%)	57 (100%) (93.7%; 100%)	57 (100%) (93.7%; 100%)
Q13	Dysphagia - Dysphonia	57 (100%) (93.7%; 100%)	57 (100%) (93.7%; 100%)	57 (100%) (93.7%; 100%)	57 (100%) (93.7%; 100%)	57 (100%) (93.7%; 100%)
Q14	Respiratory Allergy	57 (100%) (93.7%; 100%)	57 (100%) (93.7%; 100%)	57 (100%) (93.7%; 100%)	57 (100%) (93.7%; 100%)	57 (100%) (93.7%; 100%)
Q15	Patient Safety	57 (100%) (93.7%; 100%)	57 (100%) (93.7%; 100%)	57 (100%) (93.7%; 100%)	57 (100%) (93.7%; 100%)	56 (98.2%) (90.7%; 99.7%)
	Questions	Accuracy	Reproducibility	Reliability	Applicability	Relevance
Q16	Use of Resources	55 (96.5%) (88.1%; 99.0%)	55 (96.5%) (88.1%; 99.0%)	55 (96.5%) (88.1%; 99.0%)	55 (96.5%) (88.1%; 99.0%)	54 (94.7%) (85.6%; 98.2%)
Q17	Ability to Evaluate and Investigate	56 (98.2%) (90.7%; 99.7%)	55 (96.5%) (88.1%; 99.0%)	56 (98.2%) (90.7%; 99.7%)	55 (96.5%) (88.1%; 99.0%)	54 (94.7%) (85.6%; 98.2%)
Q18	Professionalism	57 (100%) (93.7%; 100%)	56 (98.2%) (90.7%; 99.7%)	57 (100%) (93.7%; 100%)	57 (100%) (93.7%; 100%)	56 (98.2%) (90.7%; 99.7%)
Q19	Interpersonal Communication	56 (98.2%) (90.7%; 99.7%)	56 (98.2%) (90.7%; 99.7%)	56 (98.2%) (90.7%; 99.7%)	57 (100%) (93.7%; 100%)	55 (96.5%) (88.1%; 99.0%)

Source: authors (2021)

[Table t3]

**Table 3 t3:** Instrument assessment according to otolaryngologists across the country.

Evaluated criterion	Average of responses
Applicable	99.25%
Accurate	99.50%
Reproducible	98.60%
Reliable	93.84%
Relevant	93.15%
General	96.86%

Source: authors (2021).

When analyzing the results, the instrument was considered applicable in 99.25% of the responses, reproducible in 98.6%, and relevant in relation to the topics addressed in 93.15%, suggesting that the instrument can be applied in training services in otolaryngology as a form of continuous and serial assessment. Regarding reliability, the questionnaire had 93.84% acceptance, suggesting that the instrument’s respondents agree that the evaluated items are addressed correctly. Regarding accuracy (99.5%) the research instrument had an excellent acceptance, which shows that the authors’ choice to include all subspecialties in the experts’ group was correct. When an item is considered accurate, it translates reality and can be regarded as true.

[Fig ch1]

**Graph 1 ch1:**
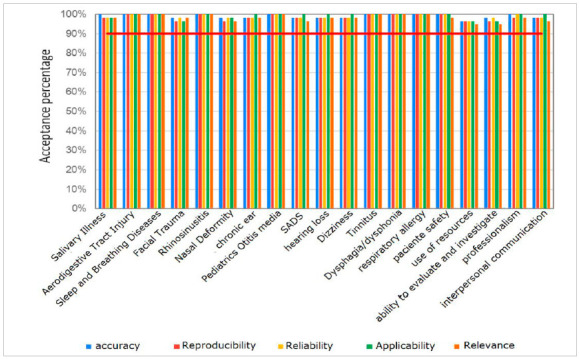
Percentages of Adequacy of Questions by Category.

In summary, 96.86% agreed that the developed instrument represents the reality of the otolaryngologist’s training, for a 3-year training period.

## DISCUSSION

For the original instruments to be valid in realities different from the ones they were created, they must not only be literally translated, but also undergo rigorous and systematic adaptation to maintain their validity and concepts across different cultures^10^. 

One of the suggested adaptations to the Brazilian reality was that the development milestones of the created instrument be evaluated at the end of the first six months of residency, at the end of the first year, at the end of the second year, and at the end of the third year, while the original instrument suggests that the evaluations be annual.

During the first training year, two evaluations are foreseen, as the otolaryngology resident is already a trained doctor, that is, he/she must present some basic concepts in evaluation, diagnosis, and treatment in otolaryngology.

The first assessment takes place at the end of the sixth month of the first year and has a diagnostic function, as physicians who graduated in different services may have developed different skills and abilities, however some basic technical and theoretical skills must have been developed during initial training. In addition to confirming the previously developed skills, at the end of the first 6 months, the otolaryngology resident should already be showing improving knowledge of the most prevalent diseases in the specialty and developing care for the preoperative preparation and postoperative management of the same diseases^11^.

The following assessments are annual and of a formative nature, as they accompany the acquisition of skills, which become more complex, from a theoretical and from a technical point of view, as the residency progresses. Regarding the structure of the original instrument, we decided to keep it, since it clearly and objectively presents the paths to be followed for the acquisition of skills in each of the topics covered, thus facilitating their evaluation.

After the translation, synthesis, and back-translation steps, the instrument was adapted for evaluation by eight experts. The panel of experts was composed of specialists in the studied content. Experts are professionals who publish or work in the studied area, this selection being essential to determine whether an instrument is well constructed and adequate^12^. 

When choosing experts, we ensured that each of the subspecialties of otolaryngology (rhinology, otoneurology, facial plastic surgery, phoniatrics, laryngology, head and neck surgery, pediatric otolaryngology, otology, and sleep medicine) was included as a subspecialty of at least one of the experts.

The fact that each of the experts had at least one of the subspecialties was relevant, since all items could be evaluated in a judicious and rigorous manner, thus not leaving any forgotten topic. The observations of all experts were cataloged and divided into each of the 17 items.

At the end of the questionnaire, there was a field, where the experts should globally evaluate the instrument, in which there were two questions. The first asked if “the concept was adequately covered”, and the second, if “all items were included”. In the first question, all experts answered yes, that all concepts had been covered. In the second, all but one answered yes. The expert’s negative response was due to the belief that two otolaryngological subtopics were missing: dizziness and tinnitus.

When reviewing the competences required by ABORLCCF, competences were identified referring to these two items that were not contemplated in the original instrument. Thus, two new items were created, one for dizziness and the other for tinnitus, with all the skills required by the ABORLCCF, and added to the first instrument, resulting in a final instrument with 19 items.

After the final analysis by experts, the instrument was sent for evaluation by otolaryngologists from all subspecialties involved in resident training. The final stage of the adaptation process is the pilot, where the objective is to assess the acceptance of the population that will use the instrument; ideally between 30 and 40 people should be tested^2^. Fifty-seven otolaryngologists answered the questionnaire.

The analyses of the responses of experts and specialists showed that the instrument can be used as an important tool for formative evaluation and should be widely applied to determine which training milestones were achieved and thus guide both residents and preceptors on the development status of each of the skills required during the training of specialists in otolaryngology.

The instrument must be constantly updated and restructured, so that it can always be in line with current treatments, surgical techniques, and teaching and learning processes.

## CONCLUSION

The work concluded all the steps proposed for the cross-cultural adaptation, faithful to the original instrument, thus being considered valid for use in Brazil.
